# Changes in acid–base and ion balance during exercise in normoxia and normobaric hypoxia

**DOI:** 10.1007/s00421-017-3712-z

**Published:** 2017-09-15

**Authors:** Olaf Lühker, Marc Moritz Berger, Alexander Pohlmann, Lorenz Hotz, Tilmann Gruhlke, Marcel Hochreiter

**Affiliations:** 1 Department of Anesthesiology, University Medical Centre Groningen, Groningen, The Netherlands; 2 Department of Anesthesiology, University Hospital Heidelberg, University of Heidelberg, Im Neuenheimer Feld 110, 69120 Heidelberg, Germany; 3 Division of Sports Medicine, Department of Internal Medicine VII, University of Heidelberg, Heidelberg, Germany; 4 Department of Anesthesiology, Perioperative and General Critical Care Medicine, University Hospital Salzburg, Paracelsus Medical University, Salzburg, Austria

**Keywords:** Acid–base balance, Exercise, Hypoxia, Lactate, Metabolic acidosis, Respiratory alkalosis

## Abstract

**Purpose:**

Both exercise and hypoxia cause complex changes in acid–base homeostasis. The aim of the present study was to investigate whether during intense physical exercise in normoxia and hypoxia, the modified physicochemical approach offers a better understanding of the changes in acid–base homeostasis than the traditional Henderson–Hasselbalch approach.

**Methods:**

In this prospective, randomized, crossover trial, 19 healthy males completed an exercise test until voluntary fatigue on a bicycle ergometer on two different study days, once during normoxia and once during normobaric hypoxia (12% oxygen, equivalent to an altitude of 4500 m). Arterial blood gases were sampled during and after the exercise test and analysed according to the modified physicochemical and Henderson–Hasselbalch approach, respectively.

**Results:**

Peak power output decreased from 287 ± 9 Watts in normoxia to 213 ± 6 Watts in hypoxia (−26%, *P* < 0.001). Exercise decreased arterial pH to 7.21 ± 0.01 and 7.27 ± 0.02 (*P* < 0.001) during normoxia and hypoxia, respectively, and increased plasma lactate to 16.8 ± 0.8 and 17.5 ± 0.9 mmol/l (*P* < 0.001). While the Henderson–Hasselbalch approach identified lactate as main factor responsible for the non-respiratory acidosis, the modified physicochemical approach additionally identified strong ions (i.e. plasma electrolytes, organic acid ions) and non-volatile weak acids (i.e. albumin, phosphate ion species) as important contributors.

**Conclusions:**

The Henderson–Hasselbalch approach might serve as basis for screening acid–base disturbances, but the modified physicochemical approach offers more detailed insights into the complex changes in acid–base status during exercise in normoxia and hypoxia, respectively.

**Electronic supplementary material:**

The online version of this article (doi:10.1007/s00421-017-3712-z) contains supplementary material, which is available to authorized users.

## Introduction

Intense muscular exercise induces multiple and simultaneously occurring chemical, physical and physiological reactions that serve to prevent excessive changes within and outside the cells. Still, the formation of lactate (Lac^−^), carbon dioxide (CO_2_) and hydrogen ions (H^+^) causes intracellular acidosis. The accumulation of Lac^−^ and H^+^ results mainly from intracellular glycolysis, while the formation of CO_2_ results from increased mitochondrial production and titration of bicarbonate and non-bicarbonate compounds (Iwato et al. 1993; Lindinger et al. 1995; Stickland et al. 2013). H^+^ and Lac^−^ are released into extracellular fluid, mainly via monocarboxylate transporters (Gladden 2004; Goodwin et al. 2007). The increased muscle activity during exercise causes repetitive depolarisations and repolarisations of the muscle cells/sarcolemma, leading to a concomitant release of potassium (K^+^) from the cell into extracellular fluid (Lindinger et al. 1992; Cairns and Lindinger 2008). The physiological response to these processes consists of a rapid and intense increase in minute ventilation. The increase in ventilation serves to match the increased oxygen demand on the one side and to remove additional CO_2_ via the lungs on the other side (Stringer et al. 1992; Lindinger and Heigenhauser 2012; Stickland et al. 2013; Wasserman et al. 2014). Hypoxia is another stimulus known to cause a rapid and large increase in minute ventilation (Bernardi et al. 2006). The result is an increased CO_2_ removal leading to respiratory alkalosis. When exercise and hypoxia are combined both the exercise-induced metabolic acidosis and the hypoxia-induced respiratory alkalosis cause even more complex changes in acid–base homeostasis.

The interdependency between changes of acid–base homeostasis and ventilation is characterized by the Henderson–Hasselbalch equation (Severinghaus and Astrup 1985; Schlichtig 1997). Together with the base excess concept that was first described by Singer and Hastings (1948), the Henderson–Hasselbalch equation serves as the basis for the traditional approach in the description of acid–base disorders (Siggaard-Andersen 1977; Morgan et al. 2000). This approach has been applied by numerous studies and in different settings to examine acid–base changes that occur upon physical exercise in normoxia and hypoxia (Medbø and Sejersted 1985; Lindinger et al. 1992; Stringer et al. 1992; Wasserman et al. 1997). However, growing evidence suggests that alternative approaches may offer better insights into non-respiratory changes of acid–base homeostasis and the complex interplay of compensating factors (Kellum 2005; Sheldon and Ali 2015).

In 1978, Stewart introduced an alternative approach that aimed to account for the role of strong ions, weak acids, CO_2_ and water itself in the regulation of H^+^-concentration (Stewart 1978, 1983; Weinstein et al. 1991; Figge 2009). This approach is now referred to as the physicochemical approach or Stewart approach to acid–base balance. However, its acceptance has been limited because it requires a complicated set of calculations, limiting the applicability of this approach in routine clinical practice (Weinstein et al. 1991; Magder and Emami 2015; Story 2016). Several modifications of the original Stewart approach have been evaluated in clinical and experimental settings over the last years (Kellum et al. 1995; Skellett et al. 2000; Durward and Murdoch 2003). For some clinical applications, these modifications allow an easier and more applicable way to characterize the acid–base status than the original Stewart approach.

The aim of the present study was to characterize the regulation of acid–base status during intense physical exercise in normoxia and normobaric hypoxia (FiO_2_ = 0.12, corresponding to an altitude of 4500 m above sea level) by use of the modified physicochemical approach (Figge et al. 1991, 1992; Corey 2003; Morgan 2009, 2011) and the traditional Henderson–Hasselbalch approach.

## Methods

The study was conducted in accordance with the Declaration of Helsinki and its current amendments and was approved by the Ethics Committee of the Medical Faculty of the University of Heidelberg (approval number S-522/2012). After being familiarized with the study protocol and providing written informed consent, 22 male healthy individuals were enrolled in the study. Three participants did not complete the second study day so that the analysis is based on the data set of 19 subjects (Table 1). None of the participants was exposed to altitudes >2000 m within 30 days before the study and during the study period. No participant took regular medications and none had a pre-existing cardiovascular or pulmonary disease. All subjects were encouraged to restrain from alcohol and exercise on the day prior to the study days. During the study days the participants received standardized food and beverages.

**Table 1:** Characteristics of the study participants

Number of participants	19
Age (years)	36 ± 3
Height (cm)	178 ± 2
Weight (kg)	74.5 ± 2
Body mass index (kg/m^2^)	23.6 ± 0.4
Resting heart rate (beats/min)	71 ± 3
Resting systolic blood pressure (mmHg)	137 ± 3
Resting diastolic blood pressure (mmHg)	88 ± 2
Training per week (h)	7.9 ± 1.0
Haemoglobin (g/dl)	15.0 ± 0.8

Data were assessed during the pre-examination 2–4 weeks prior to the first study day. Values are given as total numbers and mean ± SEM, respectively

### General procedures

On two different study days the participants performed an exercise test on a bicycle ergometer (Lode Medical Technology, Ergometrics 900, Groningen, The Netherlands) in seating position until voluntary fatigue. The workload for the exercise test started at 50 W and was increased by 50 W every 3 min. This type of exercise test was chosen to evoke a significant excursion of the acid–base status. To further exaggerate the oxygen-dependent changes in acid–base status and ventilation the exercise test was not only performed in normoxia (FiO_2_: 0.21) but also in normobaric hypoxia (FiO_2_: 0.12; equivalent to an altitude of 4500 m above sea level) achieved by admixture of nitrogen (System Linde Gas, Pullach, Germany). The sequence of exposure to normoxia and hypoxia was randomized, and both the participants and investigators were blinded with respect to the ambient oxygen concentration.

Prior to the exercise test, arterial vascular access was obtained by catheterizing the radial artery of the non-dominant hand with a 20 G intravascular catheter (Braun, Melsungen, Germany). Catheter patency was maintained with a pressurized flush system of normal saline. At each blood sampling, the first 3 ml of the probe was discarded to prevent contamination of the blood with saline. Blood samples were taken at T0 = prior to exercising, T100 = 6 min of exercise, equivalent to 100 W, T200 = 12 min of exercise, equivalent to 200 W, TTerm = termination of exercise when voluntary fatigue was reached and T6post = 6 min after termination of exercise. Timing of blood sampling during the exercise test followed the incremental increase of work load, while T6post was chosen arbitrarily as a time point during the recovery phase. Heart rate was continuously monitored via an electrocardiogram.

### Analyses of blood samples

**Fig. 1:**
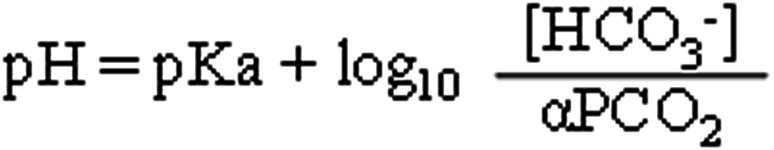
The Henderson–Hasselbalch equation. *pH* plasma pH; *pKa* negative log to base 10 of the apparent, overall dissociation constant of carbonic acid; [HCO_3_^−^ *]* plasma bicarbonate concentration; *α* solubility of carbon dioxide in blood at 37 °C;* p*CO_2_ partial pressure of carbon dioxide in blood

Immediately after drawing, part of the blood samples were analysed using a blood gas analyser (Siemens Rapid Point 500, Eschborn, Germany) for the determination of electrolytes, Lac^−^, haemoglobin, haematocrit, pH, bicarbonate (HCO_3_^−^), standard base excess (BE), and partial pressures of oxygen (PO_2_) and CO_2_ (PCO_2_). Additional blood samples were analysed by the laboratory of the University Hospital Heidelberg for albumin, total protein and phosphate. Ionized calcium and ionized magnesium were measured by ion selective electrodes (Burnett et al. 2000; Saha et al. 1996). Blood samples determined for the hospital laboratory were kept on a rotator and sent for analysis within 15 min after termination of exercise. Thus, the parameters for the traditional Henderson–Hasselbalch approach (Fig. 1) were obtained by blood gas analyses, while the parameters for the modified physicochemical approach were obtained by the combination of blood gas analyses, laboratory testing and calculations according to the following equations:

*A*_tot_^−^: 0.2627 × (albumin) + 0.0906 [(total protein − albumin)] + 2 × [*P*_*i*_].

SID_app_: [Na^+^] + [K^+^] + [Ca^++^] + [Mg^++^] − [Cl^−^] − [Lac^−^].

SID_eff_: 2.46 × 10^−8^ × PCO_2_/10^−pH^ + (albumin) × 0.1 × (0.123 × pH − 0.631) + (*P*_*i*_) × (0.309 × pH − 0.469).

SID_inorganic_: [Na^+^] + [K^+^] + [Ca^++^] + [Mg^++^] − [Cl^−^].

SIG: SID_app_ − SID_eff_.

ΔpV (%): 100 × (Hb_pre_/Hb_post_) × (100-Hct_post_)/((100 − Hct_pre_) − 1).

with *A*_tot_^−^ = net charge of non-volatile weak acids, Hb = haemoglobin, Hct = haematocrit, *P*_*i*_ = phosphate, SID_app_ = apparent strong ion difference, SID_eff_ = effective strong ion difference, SIG = strong ion gap, ΔPV = plasma volume change; electrolytes, *P*_*i*_ and Lac^−^ given in mmol/l, Hb in g/dl, Hct in % and albumin and total protein given in g/l. *A*_tot_^−^ was calculated according to Lloyd (2004). SID_app_ represents the net charge of strong ions and [Lac^−^] according to the original Stewart approach. SID_eff_ is an approximation of the net charge of the main volatile and non-volatile weak acids in plasma. It was introduced by Kellum et al. (Kellum et al. 1995). The SIG is calculated from SID_app_ and SID_eff_. These calculations are referred to as the modified physicochemical approach. SID_inorganic_ was calculated according to Noritomi et al. (2009). As very high [Lac^−^] values have a strong impact on SID_app_, calculating SID_inorganic_ allows a more detailed view of the composition of SID_app_. ΔPV was calculated according to Novosadova (1977).

### Statistics

Normal distribution of the data was tested using the Kolmogorov–Smirnov test. Data obtained periodically throughout the experiment, such as the parameters obtained for blood gas analyses and laboratory testing, were analysed by two-way repeated measures analysis of variance if they were normally distributed or by Friedman repeated measures analysis of variance on ranks if the data were not normally distributed. Pairwise multiple comparison procedures were made using Student–Newman–Keuls test if the overall test was significant. The relationship between pairs of variables was expressed with the Pearson’s correlation coefficient. Data are expressed as mean values ± SEM and box-plot diagrams, respectively. The plots show the median, 10th, 25th, 75th and 90th percentiles as vertical boxes with error bars. A *P* value of ≤0.05 was considered significant. Statistics were performed using the SigmaStat^®^ software package (SPSS Inc., Chicago, IL, USA).

## Results

Three participants did not complete both study days. One participant developed ST-segment depression on the electrocardiogram during the first study day in normoxia and was excluded, and another developed lower extremity injury and could not attend the second study day. A third participant did not attend the second study day due to personal reasons. Thus, the analysis is based on the complete dataset of 19 participants. The anthropometric data of these 19 subjects are shown in Table 1.

### Peak power output and heart rate

Peak power output on the bicycle ergometer was 287 ± 9 W in normoxia and 213 ± 6 W in hypoxia (−26%, *P* < 0.001). The corresponding weight-adjusted power output showed a decrease from 3.9 ± 0.2 W/kg in normoxia to 2.9 ± 0.1 W/kg in hypoxia (−26%, *P* < 0.001). Maximal heart rate during exercise was 192 ± 3 beats/min in normoxia and decreased to 180 ± 3 beats/min in hypoxia (*P* < 0.001).

### Arterial PO_2_

Arterial PO_2_ was 95 ± 2 mmHg at normoxic rest and decreased to 91 ± 2 mmHg at the level of peak work intensity (*P* < 0.001). During recovery arterial PO_2_ increased again, reaching 101 ± 2 mmHg at the end of the recovery phase (*P* < 0.001 versus rest). In hypoxia arterial, PO_2_ was significantly lower compared to normoxia at both rest (44 ± 2 mmHg; *P* < 0.001 versus normoxia) and at the level of peak work intensity (43 ± 1 mmHg; *P* < 0.001 versus normoxia).

### Parameters of the traditional Henderson–Hasselbalch approach and blood gas analysis

**Fig. 2:**
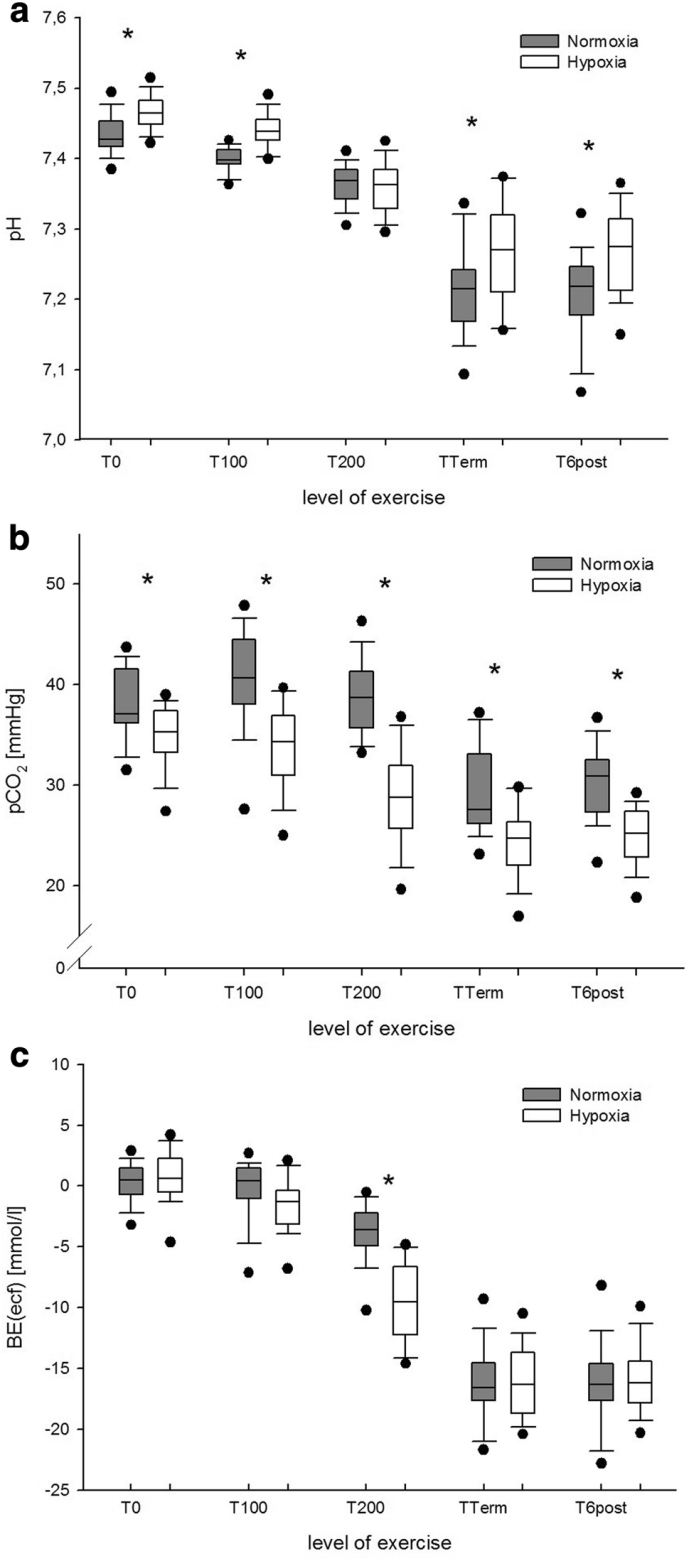
**a** Arterial pH, **b** arterial PCO_2_, and **c** arterial base excess (BE) at rest and during exercise in normoxia (grey boxplots) and hypoxia (white boxplots). *P < 0.001 for normoxia versus hypoxia at the same level of exercise

**Fig. 3:**
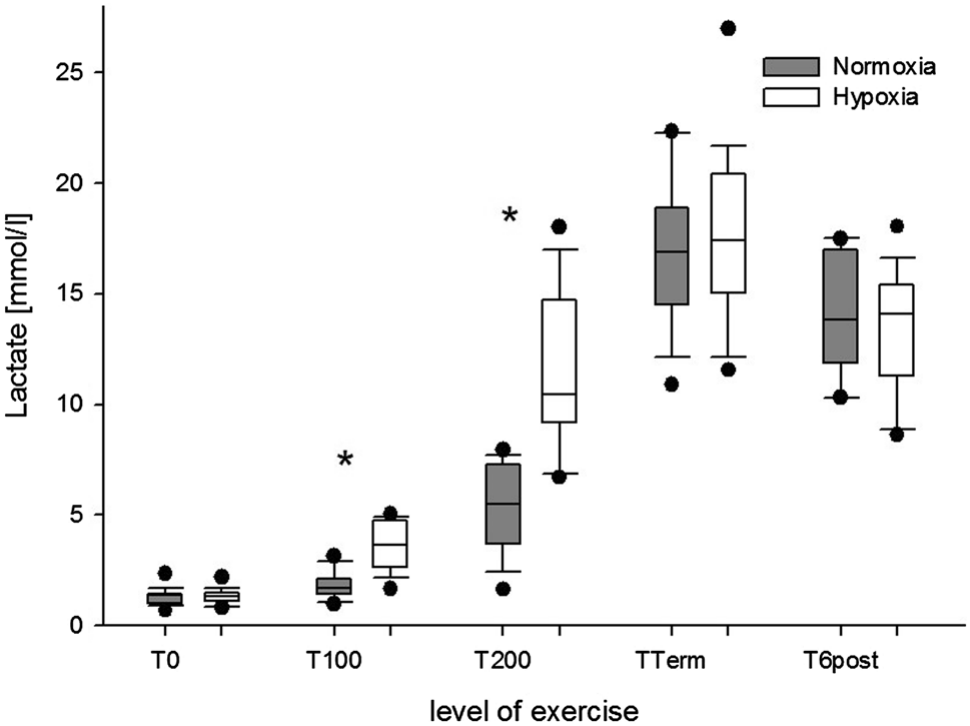
Arterial lactate concentrations at rest and during exercise in normoxia (grey boxplots) and hypoxia (white boxplots). *P < 0.001 for normoxia versus hypoxia at the same level of exercise

The changes in pH, PCO_2_ and BE that were observed during normoxic and hypoxic exercise are displayed in Fig. 2. The corresponding changes in plasma Lac^−^ are shown in Fig. 3.

### Parameters of the modified physicochemical approach

**Fig. 4:**
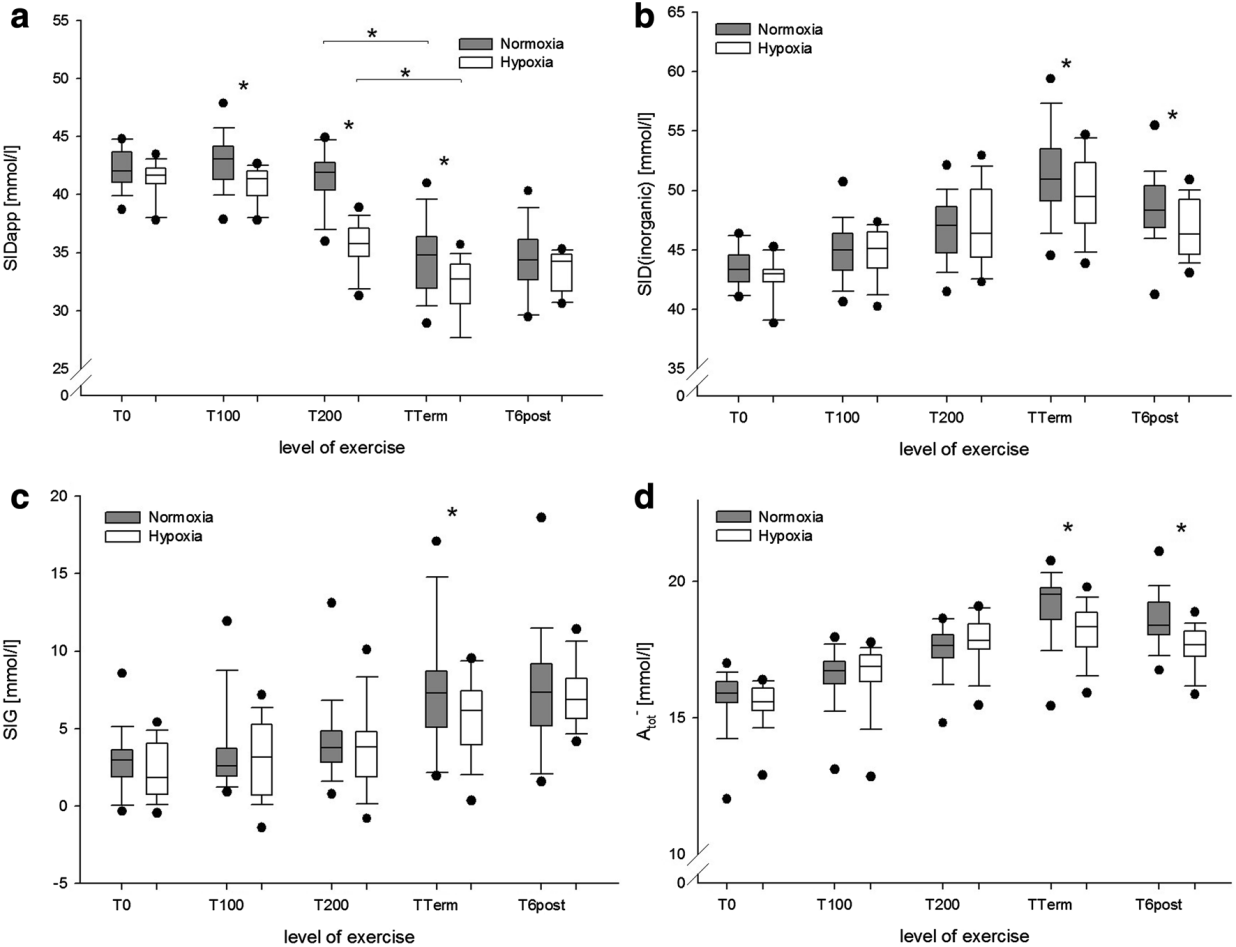
**a** Apparent strong ion difference (SID_app_), **b** inorganic strong ion difference (SID_inorganic_), **c** strong ion gap (SIG), and net charge of non-volatile weak acids (*A*_tot_^−^) at rest and during exercise in normoxia (grey boxplots) and hypoxia (white boxplots). *P < 0.001 for normoxia versus hypoxia at the same level of exercise

SID_eff_ decreased during exercise, showing significantly lower values in hypoxia, when compared to normoxia (*P* = 0.002, not shown). Lowest values were observed at peak exercise intensity (27.22 ± 2.10 mmol/l in normoxia versus 26.29 ± 2.03 mmol/l in hypoxia; *P* < 0.001). Changes in SID_app_ are shown in Fig. 4a. Because severe hyperlactatemia could have masked the changes of inorganic electrolytes, the inorganic strong ion difference (SID_inorganic_) was calculated to determine the net effects of strong cations and anions (Fig. 4b). Figure 4c shows the SIG, which is the difference of SID_app_ and SID_eff_ and was calculated to identify non-volatile acidifying or alkalinizing charges. Changes in *A*_tot_^−^ are shown in Fig. 4d.

### Plasma volume, haematocrit and albumin

**Fig. 5:**
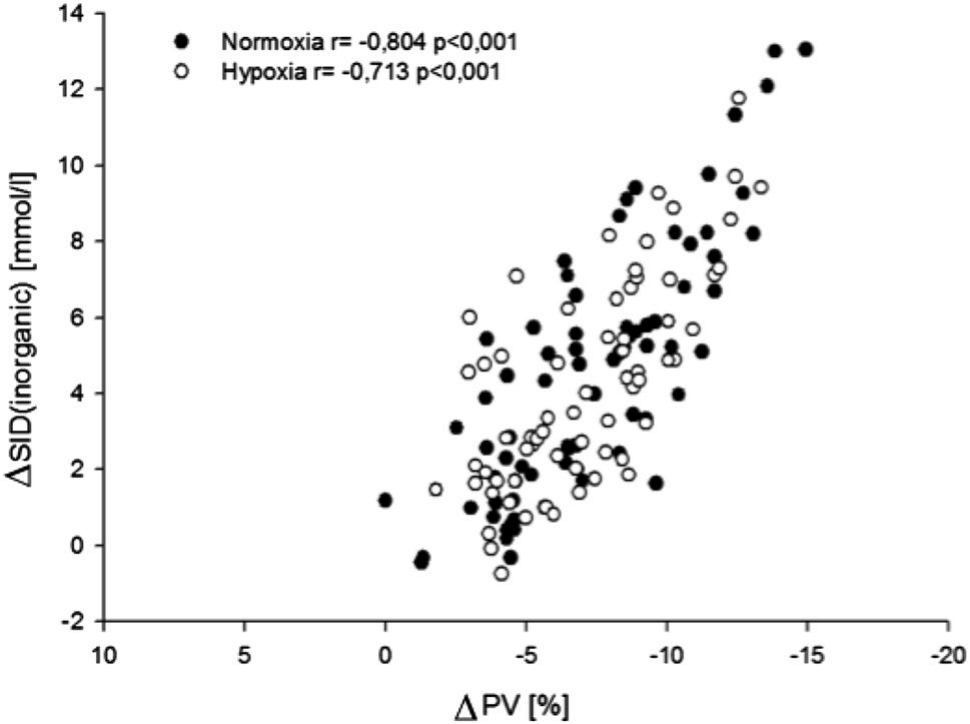
Correlation between changes in SID_inorganic_ and changes in plasma volume in normoxia (black dots) and hypoxia (white dots)

Plasma volume decreased during exercise, reaching a nadir in both groups at the level of peak exercise intensity (−11 ± 2% in normoxia, −10 ± 4% in hypoxia, *P* = 0.288 for normoxia versus hypoxia). At T100 (−3.9 ± 1.8% in normoxia versus −5.5 ± 2.8% in hypoxia) and T200 (−6.2 ± 1.8% in normoxia versus −9.1 ± 3.6% in hypoxia), the decrease in plasma volume was significantly greater in hypoxia than in normoxia (*P* < 0.001). Plasma volume increased significantly during the recovery phase (*P* < 0.001 for TTerm versus T6post in both normoxia and hypoxia, respectively), without a difference between both study conditions. There was a significant correlation between the changes in plasma volume and the changes in SID_inorganic_ (*P* < 0.001; Fig. 5).

In normoxia, haematocrit increased from 0.47 ± 0.02 at rest to 0.52 ± 0.03 at the level of peak exercise intensity (*P* < 0.001). In hypoxia, haematocrit increased from 0.47 ± 0.03 to 0.52 ± 0.03 (*P* < 0.001). There was no significant difference between both groups at TTerm (*P* = 0.619). Compared to hypoxia haematocrit was significantly lower in normoxia at T100 and T200 (both *P* < 0.001). During the 6-min recovery phase, haematocrit decreased significantly when compared to the level of peak work intensity in both normoxia and hypoxia, but remained elevated when compared to resting values (all *P* < 0.001; not shown).

The albumin concentration increased significantly during exercise from 43.38 ± 3.22 g/l at rest to 50.36 ± 3.25 g/l at the level of peak exercise intensity in normoxia, and from 43.24 ± 2.25 to 49.52 ± 2.67 g/l in hypoxia (both *P* < 0.001). There was no significant difference in albumin between normoxia and hypoxia.

### Strong ions and phosphate

The plasma concentrations of the measured strong ions ([Na^+^], [K^+^], [Ca^2+^], [Mg^2+^], [Cl^−^]) and phosphate ion species ([P_i_]) are shown in Table 2. Changes in the plasma concentrations of strong ions and [P_i_] were calculated as difference between resting and peak values and are shown in Fig. 1 of the online supplement. [K^+^] and [P_i_] showed greater changes in normoxia than in hypoxia (*P* < 0.05 for normoxia versus hypoxia).

**Table 2:** Plasma strong ion and phosphate concentrations in normoxia (*N*) and hypoxia (*H*)

	Rest			T100			T200			TTerm			T6post		
	*N*	*H*	*P*	*N*	*H*	*P*	*N*	*H*	*P*	*N*	*H*	*P*	*N*	*H*	*P*
[Na^+^]	139.2 ± 0.4	138.3 ± 0.4	ns	140.8 ± 0.4	140.2 ± 0.4	ns	143.2 ± 0.6	143.9 ± 0.9	*	148.8 ± 0.7	146.5 ± 0.6	**	143.1 ± 0.4	140.9 ± 0.5	**
[K^+^]	4 ± 0.05	3.9 ± 0.03	ns	4.5 ± 0.05	4.4 ± 0.04	ns	5 ± 0.06	5.4 ± 0.1	**	6.1 ± 0.1	5.6 ± 0.1	*	3.9 ± 0.07	3.8 ± 0.06	ns
[Cl^−^]	103.3 ± 0.4	103.1 ± 0.4	ns	104.2 ± 0.4	103.7 ± 0.4	ns	105.1 ± 0.5	106 ± 0.4	*	107.5 ± 0.5	106.5 ± 0.4	*	102.3 ± 0.4	101.9 ± 0.4	ns
[Ca^2+^]	1.2 ± 0.01	1.2 ± 0.01	ns	1.23 ± 0.01	1.22 ± 0.01	ns	1.26 ± 0.01	1.25 ± 0.01	ns	1.35 ± 0.01	1.31 ± 0.01	**	1.26 ± 0.01	1.24 ± 0.01	*
[Mg2+]	0.83 ± 0.02	0.84 ± 0.02	ns	0.86 ± 0.02	0.87 ± 0.02	ns	0.88 ± 0.02	0.91 ± 0.02	ns	0.95 ± 0.03	0.94 ± 0.02	ns	0.90 ± 0.02	0.90 ± 0.02	ns
[P_i_]	0.99 ± 0.03	0.91 ± 0.03	ns	1.05 ± 0.03	0.92 ± 0.03	*	1.26 ± 0.03	1.13 ± 0.04	*	1.56 ± 0.04	1.21 ± 0.04	**	1.41 ± 0.04	1.12 ± 0.03	**

All values in mmol/l and shown as mean ± SEM

*P < 0.05; **P < 0.001

## Discussion

The present study aimed to characterize the effect of exercise until fatigue on acid–base status in normoxia and normobaric hypoxia by use of the traditional Henderson–Hasselbalch approach and by application of the modified physicochemical approach (Figge et al. 1991, 1992; Kellum et al. 1995; Corey 2003; Morgan 2009, 2011). The study also aimed at characterizing whether in this situation the modified physicochemical approach offers a better understanding of the changes in acid–base homeostasis compared to the easier applicable traditional Henderson–Hasselbalch approach. These aims were achieved by analysing arterial blood samples gained during the exercise test with both approaches and by comparing the gathered information with respect to the underlying mechanisms of the observed changes. Understanding the origin and contribution of each substance to an acid–base disturbance is important for the causal treatment of acid–base disturbances.

### Traditional Henderson–Hasselbalch approach

The analysis of the parameters of the traditional Henderson–Hasselbalch approach showed that exercise significantly decreased pH, reflecting a rapid and progressive acidification of the plasma with increasing exercise intensity. One might assume that during exercise, and even earlier during hypoxic exercise, acidosis was primarily caused by Lac^−^. The Lac^−^ anion builds-up mainly in actively exercising muscle and is transported out of the cell by monocarboxylate transporters (Gladden 2004; Cairns and Lindinger 2008). A fast transport of Lac^−^ across the plasma membrane is important for muscle function and for maintaining muscle pH despite the high H^+^ and Lac^−^ load that develops during intense exercise (Kowalchuk and Scheuermann 1995; Cairns and Lindinger 2008). The present study shows that at peak exercise intensity the plasma concentration of Lac^−^ did not differ between normoxia and hypoxia. This finding is in accordance with Kato et al. who found that peak values of plasma Lac^−^ did not differ between normoxia and hypoxia (Kato et al. 2004). In hypoxia, the peak power output was reduced by ~26%, what is in line with previous studies (Fulco et al. 1998; Kato et al. 2004). This finding suggests that rather than the inspired oxygen fraction (FiO_2_) the intracellular Lac^−^ levels and transmembrane ion fluxes determined intracellular [H^+^] and led to voluntary fatigue and termination of exercise. However, which of these factors contribute the most to muscle fatigue still remains controversial (Lindinger et al. 1995; Gladden 2004; Cairns and Lindinger 2008; Morales-Alamo et al. 2015).

According to the traditional approach, BE represents a non-respiratory (metabolic) component of acid–base status. The increase in plasma Lac^−^ during exercise we observed showed a close inverse correlation with BE and HCO_3_^−^ (not shown), indicating that Lac^−^ was an important contributor to exercise-induced metabolic acidosis. However, it has previously been suggested that the exercise-induced increase in Lac^−^ is not the only contributor to exercise-induced acidosis (Sejersted et al. 1982; Lindinger et al. 1992; Lindinger and Heigenhauser 2008).

Despite the higher plasma concentrations of Lac^−^ at T100 and T200, pH values remained significantly higher in hypoxia than in normoxia. This finding indicates that in hypoxia compensating mechanisms averted a stronger plasma acidification. According to the traditional approach, the lower *P*CO_2_ values caused by hypoxia-induced hyperventilation counteracted the severity of exercise-induced metabolic acidosis. In hypoxia and at high altitude, respiratory alkalosis might be favourable (West 2000; Samaja et al. 2003; Winslow 2007; Mollard et al. 2008). However, at an exercise intensity of 200 W, pH values were about the same in normoxia and hypoxia, with *P*CO_2_ values being lower and Lac^−^ values being higher in hypoxia. These findings reflect the rapid and intense respiratory response that occurs during intense exercise (Davies et al. 1986; Stringer et al. 1992; Wasserman et al. 2014). Kato et al. showed similar changes for pH and *P*CO_2_ as in the present study (Kato et al. 2004). The authors suggested a pH-dependency of muscle Lac^−^-release which has been described for both, respiratory alkalosis and HCO_3_^−^-induced metabolic alkalosis (Davies et al. 1986; McLellan et al. 1988; LeBlanc et al. 2002). Thus, alkalinizing plasma by hyperventilation could have modified the Lac^−^-shift from exercising muscle into circulating blood (Davies et al. 1986; Lindinger et al. 1992; Kato et al. 2004).

However, while the traditional approach sufficiently describes the changes of some variables many other aspects of acid–base homeostasis that are considered in the modified physicochemical approach are not taken into account.

### Modified physicochemical approach

According to the Stewart approach, acid–base status is determined by three independent variables, i.e. *P*CO_2_, SID and *A*_tot_^−^. Only if at least one of these variables changes, the dependent variables, i.e. pH, HCO_3_^−^ and CO_3_^2−^, may be altered.

In the present study, SID_app_ decreased during both normoxic and hypoxic exercise, reflecting metabolic acidosis, of which Lac^−^ seemed the main contributor. Simultaneously, plasma concentrations of inorganic strong ions increased, ultimately leading to an increase in SID_inorganic_, thus alleviating the acidifying effect of Lac^−^. However, quantitatively, the increase in SID_inorganic_ could not fully compensate for the increase in Lac^−^ as reflected by the decrease in pH. Notably, while at the level of peak exercise intensity, Lac^−^ values were about the same in normoxia and hypoxia, SID_inorganic_ and SID_app_ were significantly higher in normoxia. This indicates that during normoxia a more pronounced alkalinizing process had occurred, ultimately contributing to higher pH values. In combination with the higher *P*CO_2_ values that were observed in normoxia, the results indicate that in situations of insufficient respiratory compensation, non-respiratory mechanisms, e.g. ion-shifts, significantly affect acid–base regulation.

Another independent variable that affects pH as well as other dependent variables of the Stewart approach is *A*_tot_^−^. Main contributors to *A*_tot_^−^ are albumin, globulins and [P_i_]. Several different formulas have been applied to calculate *A*_tot_^−^ in a clinically feasible way (Figge et al. 1991; Constable 2001; Staempfli and Constable 2003; Lloyd 2004). In the present study, *A*_tot_^−^ was calculated according to the equation from Lloyd (2004). The results show that both an increase in albumin and [P_i_] caused a significant increase in *A*_tot_^−^. Although albumin concentrations were not different between normoxia and hypoxia, the contribution of albumin to *A*_tot_^−^ was ~20% higher in hypoxia. This might be explained by the almost linear relationship between the ionic charge of albumin and plasma pH (Figge et al. 1991, 1992; Fogh-Andersen et al. 1993; Figge 2009). Because pH values were higher in hypoxia, the negative ionic charge of albumin had increased and in turn elevated the albumin fraction of *A*_tot_^−^.

The contribution of [P_i_] to *A*_tot_^−^ was ~30% greater in normoxia than in hypoxia. The phosphoric ionic system is not as pH dependent as albumin. Because of their trivalent structure, the phosphorous ions have different dissociation equilibria, whose titration curves follow a triphasic course. Therefore, exercise-induced changes in the concentration of [P_i_] rather than pH changes determined the contribution of [P_i_] to *A*_tot_^−^ in the present study.

Evidence suggests that beside albumin, globulines and [P_i_], also other weak and strong ions alter acid–base homeostasis during exercise (Forni et al. 2006; McKinnon et al. 2008). Particularly, amino acids, intermediates of the Krebs cycle, tricarboxylic acids and ammonia are released into the blood and may affect acid–base balance (Sewell et al. 1994; Wagenmakers 1998; Casas et al. 2001; Kato et al. 2004). As most of these substances are organic acids, it is plausible that their anions also contributed to both the observed increase in *A*_tot_^−^ and the decrease in SID, thus generating an additional acidifying load. It is generally accepted that determination of *A*_tot_^−^ is sufficiently precise for clinical purposes, when it is calculated from the net charge of albumin, globulines and [P_i_]. However, by applying this mathematical “shortcut”, the otherwise unmeasured anions could be missed and their contribution to acid–base behaviour remain uncertain. Based on the work of Stewart, Figge, Fencl and Mydosh (Figge et al. 1991, 1992), Kellum proposed a method to quantify unmeasured ions in the context of a modified physicochemical approach (Kellum et al. 1995), which is now referred to as the SIG. By calculating SID_app_ and SID_eff_, the remainder represents unmeasured ions that contribute to acidosis if SIG is >0, or to alkalosis if SIG results in negative values. In the present study, SIG increased significantly during exercise in normoxia and hypoxia. However, at the level of peak exercise intensity, SIG was significantly higher in normoxia, indicating a higher plasma concentration of unmeasured anions. These findings could be explained by an alkalosis-related and altered release of ammonia and organic acids into the plasma during hypoxia (Casas et al. 2001; Kato et al. 2004; McKinnon et al. 2008).

In the present study, plasma Lac^−^ increased faster in hypoxia than in normoxia. This faster release of Lac^−^ probably blunted intracellular acidosis and thus the release of organic acids and ammonia. Kato et al. reported lower plasma ammonia levels during exercise in hypoxia (FiO_2_ = 0.12) when compared to normoxia (Kato et al. 2004), while other authors showed increased ammonia levels after exercise in normoxia (Sewell et al. 1994; Casas et al. 2001). Alkalinisation of blood by hypoxia and exercise-induced respiratory alkalosis might have caused a change in transmembrane transport of ammonia and organic acids. The impact of this organic compound on acid–base homeostasis is not yet fully clarified. Several clinical and experimental studies suggest these organic compounds to be organic acids as well as ketone bodies and metabolic intermediates of the intracellular cycles of glucose and fatty acid metabolism (Forni et al. 2006; Moviat et al. 2008). McKinnon et al. investigated this compound using liquid chromatography and enzyme assays (Forni et al. 2006; McKinnon et al. 2008). They found that beside the well-known exercise-induced lactic acidosis, increased plasma concentrations of α-ketoglutarate, citrate, isocitrate and malate contributed to the acidic load (McKinnon et al. 2008). Although the SIG does not determine the origin of all contributing anions, they may be quantified and thus allow a more precise description of the acid–base changes. However, the SIG has its limitations. SIG itself represents a sum of competing acidifying or alkalinizing ions, which possibly could extinguish each other’s impact on acid–base changes. Thus, SIG allows the calculation of the net effect of unmeasured ions without describing their specific nature. Another weakness of SIG is that its calculation requires many different variables, whose errors in measurements can magnify and falsify the validity of SIG. Nevertheless, calculating SIG is more accurate than calculating the traditional anion gap (Kellum et al. 1995; Forni et al. 2006).

In the present study, exercise significantly increased plasma albumin, what is in line with previous studies that attributed this finding to an exercise-induced reduction in plasma volume (Novosadová 1977; Iwato et al. 1993; Haskell et al. 1997; Kargotich et al. 1998; Alis et al. 2015). At the level of peak exercise intensity, there was no significant difference in the albumin concentration between normoxia and hypoxia. Likewise, the reduction in plasma volume did not differ at peak exercise intensity but was significantly higher in hypoxia at T100W and T200W. These findings were paralleled by changes in haematocrit, indicating that plasma volume contraction occurred earlier during exercise in hypoxia than in normoxia, respectively.

The observed increase in albumin concentration was higher than what has previously been attributed to exercise-induced plasma volume contraction or exercise-induced albumin losses (Hansen et al. 1994; Haskell et al. 1997). However, albumin was not the only contributor to the increase in *A*_tot_^−^, as confirmed by the independent increase in SIG. In fact, plasma volume contraction could have contributed to the increase in inorganic ions (Table 2), which in turn resulted in an increase in SID_inorganic_. However, during exercise, K^+^ and Ca^2+^ are added to plasma which also additionally contributes to an increase in SID_app_ and SID_inorganic_. In fact, the degree of plasma volume contraction and the increase in SID_inorganic_ correlated well, suggesting that the exercise-induced decrease in plasma volume contributed significantly to the increase in SID_inorganic_. During exercise a complex shift of ions, water, and CO_2_ takes place between different compartments (i.e. intracellular space, interstitial space, red blood cells and plasma) which is determined by intracellular hydrolysis of phosphocreatine, glycolysis, CO_2_ production, intracellular Lac^−^ and H^+^ accumulation and release of these products and K^+^ into extracellular fluids, where RBC plays a crucial role in handling and distributing these products (Sejersted et al. 1982; Medbø and Sejersted 1985; Lindinger et al. 1992; Gladden 2004; Cairns and Lindinger 2008). Thus, these processes result from the efforts of the cell to satisfy energy demands and prevent cellular damage. With respect to the complexity of these processes and the number of physiologically active compartments, changes in plasma acid–base status are net effects and difficult to interpret in terms of cause and origin.

Regarding the very complex mechanisms of acid–base changes during exercise and hypoxia, in the present study, the modified physicochemical approach offered a more detailed and precise view on the different variables of acid–base control as did the traditional Henderson–Hasselbalch approach.

### Limitations

In the present study, a spiroergometry was not performed, which would have allowed a more detailed description of gas exchange and CO_2_ removal. Furthermore, we did not take into account the changes in oxygen saturation and pH dependency of buffer capacity, i.e. the Bohr and Haldane effect (West 1982; Böning et al. 1997; Samaja et al. 2003). As addressed above, inaccuracy of measurements could have affected the calculated data, i.e. SIG, as it requires several individual measurements of Alb, [P_i_^−^] and electrolytes.

Applying the original Stewart approach would have allowed to calculate the explicit contributions of the independent variables to changes in pH ([H^+^]) and HCO_3_^−^. However, because the Stewart approach is regarded as cumbersome and not attractive for routine clinical purposes we used the easier applicable modified Stewart approach.

The exercise test followed an incremental protocol until voluntary fatigue. This type of exercise test was chosen to evoke a significant excursion of the acid–base status and does not allow the characterization of acid–base changes during steady state conditions, which may significantly differ from the dynamic changes that we investigated.

## Conclusion

The modified physicochemical approach identified several competing acidifying and alkalinizing effects that were not detected with the traditional Hendersson–Hasselbalch approach. These techniques could considerably be implemented in routine clinical settings. Thus, the application of the modified physicochemical approach offered more precise insights into acid–base status during exercise in both normoxia and hypoxia than the traditional Henderson–Hasselbalch approach. Particularly, alterations in SID, SIG and *A*_tot_^−^ could be quantified and evaluated in terms of their impact on acid–base homeostasis.

## Electronic supplementary material

Below is the link to the electronic supplementary material.

Supplementary material 1 (TIF 24 KB)

## Abbreviations

A_tot_^−^: Non-volatile weak acids
BE: Base excess
CO_2_: Carbon dioxide
F_i_O_2_: Fraction of inspired oxygen
H^+^: Hydrogen ion
Hb: Haemoglobin
Hct: Haematocrit
HCO_3_^−^: Bicarbonate
Lac^−^: Lactate
m: Meter
PO_2_: Partial pressure of oxygen
PCO_2_: Partial pressure of carbon dioxide
Pi: Phosphate ion species
SEM: Standard error of the mean
SID_app_: Apparent strong ion difference
SID_eff_: Effective strong ion difference
SID_inorganic_: Inorganic strong ion difference
SIG: Strong ion gap
ΔPV: Change in plasma volume
