# Supplementary figures and images for: Changes in acid–base and ion balance during exercise in normoxia and normobaric hypoxia
Source: Eur J Appl Physiol. 2017 Sep 15;117(11):2251–61. doi: 10.1007/s00421-017-3712-z (PMC5640730; doi:10.1007/s00421-017-3712-z)

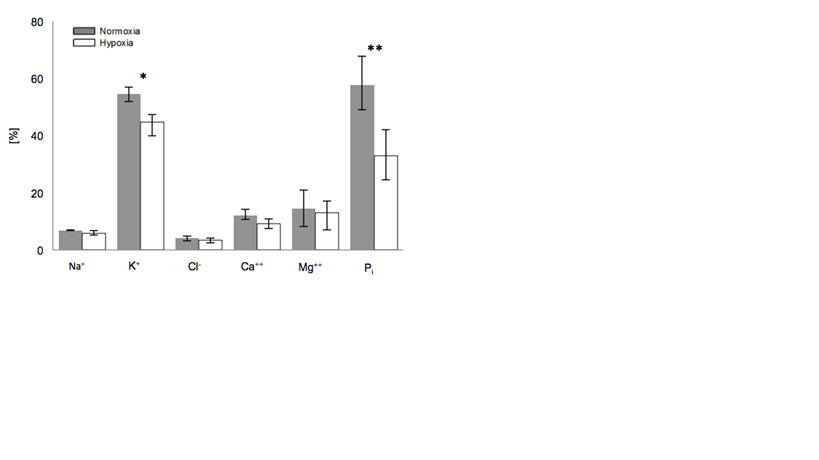

Supplement: Supplementary file 1 — Supplementary material 1 (TIF 24 KB) [file 421_2017_3712_MOESM1_ESM.tif]
